# A Biobank of Colorectal Cancer Patient-Derived Xenografts

**DOI:** 10.3390/cancers12092340

**Published:** 2020-08-19

**Authors:** Suad M. Abdirahman, Michael Christie, Adele Preaudet, Marie C. U. Burstroem, Dmitri Mouradov, Belinda Lee, Oliver M. Sieber, Tracy L. Putoczki

**Affiliations:** 1 Personalised Oncology Division, The Walter and Eliza Hall Institute of Medical Research, Parkville, Victoria 3052, Australia; abdirahman.s@wehi.edu.au (S.M.A.); christie.m@wehi.edu.au (M.C.); preaudet.a@wehi.edu.au (A.P.); burstroem.l@wehi.edu.au (M.C.U.B.); Mouradov.d@wehi.edu.au (D.M.); lee.b@wehi.edu.au (B.L.); sieber.o@wehi.edu.au (O.M.S.); 2 Department of Medical Biology, University of Melbourne, Parkville, Victoria 3010, Australia; 3 Department of Pathology, University of Melbourne, Parkville, Victoria 3010, Australia; 4 Department of Surgery, University of Melbourne, Parkville, Victoria 3010, Australia; 5 Department of Biochemistry and Molecular Biology, Monash University, Clayton, Victoria 3800, Australia

**Keywords:** colon cancer, chemotherapy, model, patient-derived, xenograft

## Abstract

Colorectal cancer (CRC) is a challenging disease, with a high mortality rate and limited effective treatment options, particularly for late-stage disease. Patient-derived xenografts (PDXs) have emerged as an informative, renewable experimental resource to model CRC architecture and biology. Here, we describe the generation of a biobank of CRC PDXs from stage I to stage IV patients. We demonstrate that PDXs within our biobank recapitulate the histopathological and mutation features of the original patient tumor. In addition, we demonstrate the utility of this resource in pre-clinical chemotherapy and targeted treatment studies, highlighting the translational potential of PDX models in the identification of new therapies that will improve the overall survival of CRC patients.

## 1. Introduction

Colorectal cancer (CRC) is the most common gastrointestinal malignancy and accounts for approximately 10% of cancer-associated mortality worldwide [1]. The clinical management of CRC depends on the stage of disease. Early stage disease often undergoes surgical resection [2]. After histological review of the resected tumor, an assessment of the lymph node involvement, perineural invasion, lymphovascular invasion, and resected margins is made, and subsequent recommendations for adjuvant therapy determined [2]. If diagnosed early, intervention is aimed at achieving cure of the disease. However, when diagnosed at late stages, there are few effective treatment options available, with most provided with palliative intent. With poor outcomes for patients with late stage disease, new models are actively being developed to enable pre-clinical studies.

Traditional pre-clinical models of CRC, including cell-lines or cell-line-derived xenografts, have been instrumental in our evolving understanding of tumor development and determining the efficacy and the mechanism of action of new therapeutic agents [3,4,5]. However, the usefulness of cell lines is hampered by clonal selection and genetic drift with models often interrogated in 2D conditions [6,7,8,9]. Misidentification and carry-over contamination of cell-lines has also been problematic [10,11,12]. To overcome these limitations and improve the accurate representation of human cancers in pre-clinical studies, patient-derived xenograft (PDX) models have emerged as a superior alternative.

PDX models were first described more than 40 years ago [13,14,15]. PDXs are generated by transplantation of fresh patient primary or metastatic tumor specimens into immunocompromised mice, bypassing the pitfall of ex vivo adaptation of monolayer cultures [16]. PDX tumors have been shown to retain the tissue architecture of the original human tumors from which they were derived, a feature often lost in cell line xenografts [16]. Given these advantages, PDX models have been established for a wide range of human epithelial cancers, including breast [17,18,19,20,21], pancreatic [22,23,24,25], lung [26,27,28,29], ovarian [30,31,32,33,34,35], and CRC [36,37,38,39,40]. The histopathological features, gene expression profiles, copy number variation, and chromosomal stability of PDX tumors are widely reported to be comparable with the original patient tumor during early passages [38,39]. Moreover, PDXs have been suggested to represent the genetic profiles of primary tumors more closely than patient-derived organoids [37]. Accordingly, PDX models have been successfully used for predicting drug sensitivity patterns of primary tumors [41].

In CRC, 5-FU (fluorouracil)-based chemotherapies are the standard-of-care for stage III and high-risk stage II patients in the adjuvant setting as well as for the treatment of advanced metastatic disease [42,43]. Patients who recur after completing 5-FU-based therapy may be classified as 5-FU resistant, while tumors progressing during the course of 5-FU based therapy may be classified as 5-FU refractory [44]. Unfortunately, the overall response rate of 5-FU in advanced CRC is limited to 10–15% [45], although this can be improved in combination with other cytotoxic agents [46] including oxaliplatin or irinotecan, in the first line setting albeit with increased toxicity [45,46]. Since resistance to 5-FU remains a major cause of failure of chemotherapy [47], model systems to better understand patient response to 5-FU are required.

Here, we describe the generation and characterization of a CRC PDX biobank derived from chemotherapy-naive stage I to stage IV tumors obtained following surgical resections, providing a platform for understanding treatment resistance, identification of new treatment opportunities, or treatment combinations for CRC patients.

## 2. Results

### 2.1. Establishment of Patient-Derived Xenografts

**Figure 1:**
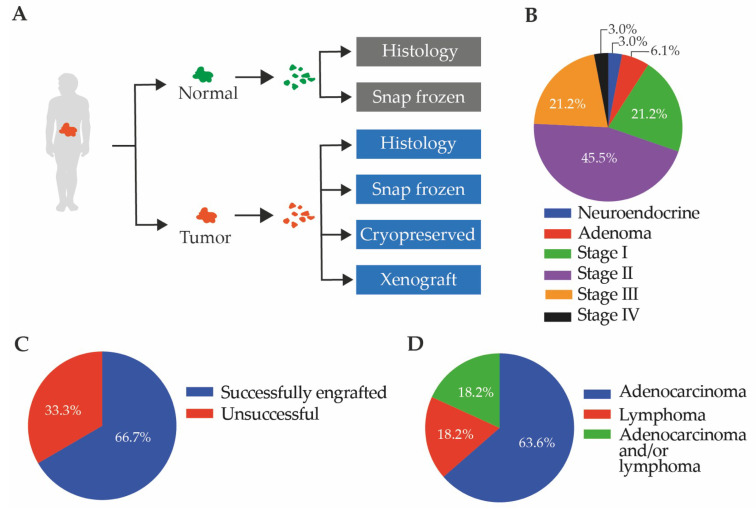
Establishment of colorectal cancer (CRC) patient-derived xenografts. (**A**) Schematic representation of the generation of a CRC biobank; (**B**) the stage of the tumors implanted into mice; (**C**) the engraftment success rate (22/33); and (**D**) the proportion of successful patient-derived xenografts (PDX) tumor engraftments that maintained only epithelial tumors (14/22; includes two adenomas), compared to lymphocytic tumors (4/22; includes one neuroendocrine tumor) or both (4/22). All epithelial tumors were included in the biobank.

Surgically resected normal colon and adjacent CRC tissue were collected from four hospitals in Melbourne, Australia (Figure S1A). In total, 33 primary chemotherapy-naïve patient-derived tumor samples were collected between 2015 and 2017 and transplanted subcutaneously into NOD scid IL2R gamma null (NSG) mice (Figure 1A). Adjacent tissue was processed for histological analysis, and additional tissue was snap frozen or cryopreserved as part of our biobank (Figure 1A). The majority (66.7%) of tumors collected were stage I/II, and the remainder (24.2%) were stage III/IV (Figure 1B) [48]. Two adenomas and one neuroendocrine tumor were also collected (Figure 1B).

PDX engraftment was deemed successful if transplanted tumors started growing within a six-month timeframe. Of the 33 primary tumors that were transplanted, 22 PDX lines were successfully generated with an overall engraftment success rate of 66.7% (22/33; Figure 1C,D). The patient-derived tumors that did not successfully engraft did not contain visible tumors cells as assessed by histopathology (PDX3, PDX32, PDX39 and PDX17; Figure S1B). Other tissues comprised large areas of stroma (PDX8, PDX9, PDX12, PDX13, PDX23, PDX33, and PDX40), which may have impacted engraftment success if predominantly stromal cells were implanted (Figure S1B).

#### 2.1.1. The Patient-Derived Xenografts Are Representative of Diverse Patient Populations

The established PDXs reflected the diversity of CRC patient clinical features, including age, gender, primary tumor location, presence of metastases at the time of diagnosis, and the differentiation state of the tumor (Table S1). PDXs were successfully derived from patients aged 18 to 86 years (median = 66 years), with the majority of patients between 50 and 80 years of age [49]. Included in our patient cohort were 14 females and 19 males, with a slightly higher incidence in males commonly reported for CRC [1]. The adenocarcinomas were histopathologically described as well-differentiated tumors (1/30; 3%), moderately differentiated (26/30; 87%), or poorly differentiated (3/30; 10%; Table S1; Table S2). The majority of primary adenocarcinomas were T3/T4 (23/30; 77%), and the remaining were T1/T2 (7/30; 23%) [50,51]. According to the primary lymph node status (pN), most patients did not present with metastasis (22/30; 73%), although a small proportion (8/30; 27%) did have evidence of metastasis to at least one lymph node.

#### 2.1.2. The CRC Patient-Derived Xenografts Successfully Underwent Serial Transplantation

**Figure 2:**
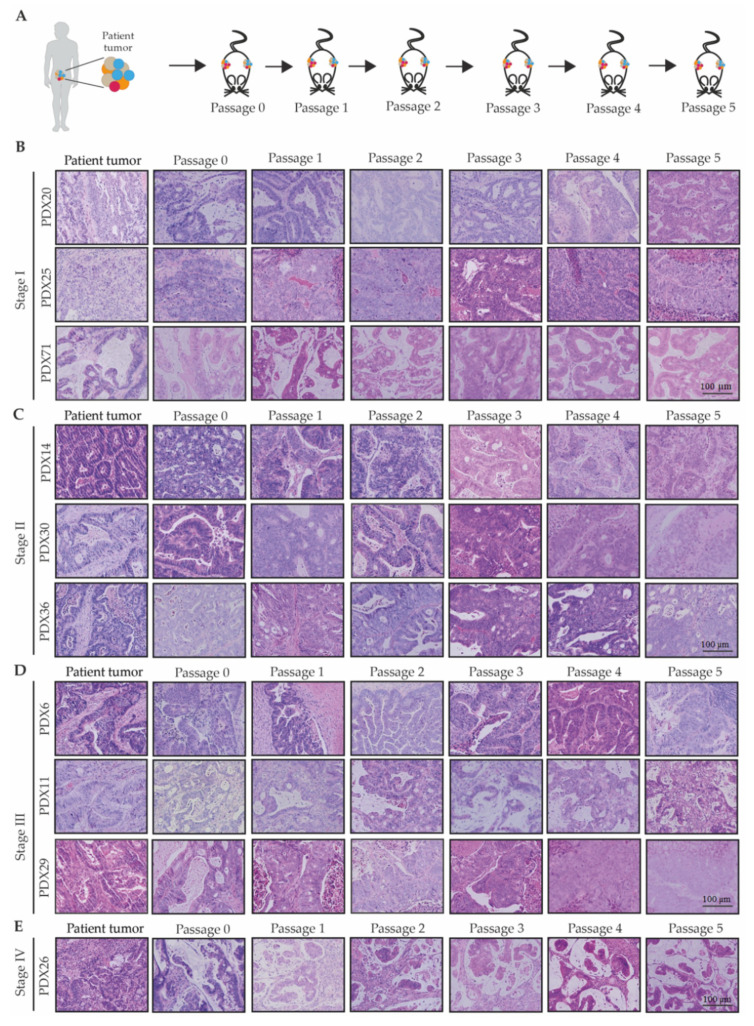
Histological characterization of CRC patient-derived xenografts. (**A**) Schematic representation of the passaging of PDXs; (**B**–**E**) representative Haematoxylin & Eosin (H&E) images of xenograft tumors from each passage. Each of the PDX lines are separated by stage: (**B**) Stage I, (**C**) Stage II, (**D**) Stage III, and (**E**) Stage IV. Scale bar for B–D is 100 µm and applies to all images.

Serial transplantation was performed for all 22 of the successfully engrafted PDXs (Figure 2A). For 10 of these PDXs, this was successfully performed for six serial passages, with the initial baseline passage denoted as passage 0 (P0; Figure 2B–E). In addition, PDX28 and PDX37 (Figure S2A) were passaged four times, and PDX1, PDX15, and PDX27 were passaged six times (Figure S2A). PDX2, the neuroendocrine tumor, was passaged five times. PDX16 could not be maintained during serial transplantation, which was likely due to the xenograft tumor being cystic and thus difficult to sample viable tumor tissue from; as a result, it was terminated at P2 (Figure S2B). A further two of the PDXs (PDX10 and PDX34), which were adenomas, were serially transplanted for two and six passages respectively (Figure S2C).

#### 2.1.3. The Patient-Derived Xenografts Maintained the Histological Features of the Patient Tumor

Most PDX tumors maintained similar histological features as the parental tumors (Figure 2B–E). However, the stromal compartment of the PDX tumors appeared decreased in volume compared to original patient tumors. However, the organization of epithelial and stromal cells relative to each other was recapitulated in the PDX tumors. This is consistent with previous reports that the epithelial cells that are of human origin can instruct the murine stromal cells to organize spatially in order to support human cancer cell growth [52].

#### 2.1.4. A Lymphocytic Phenotype Emerged for a Sub-Set of the Patient-Derived Xenografts

**Figure 3:**
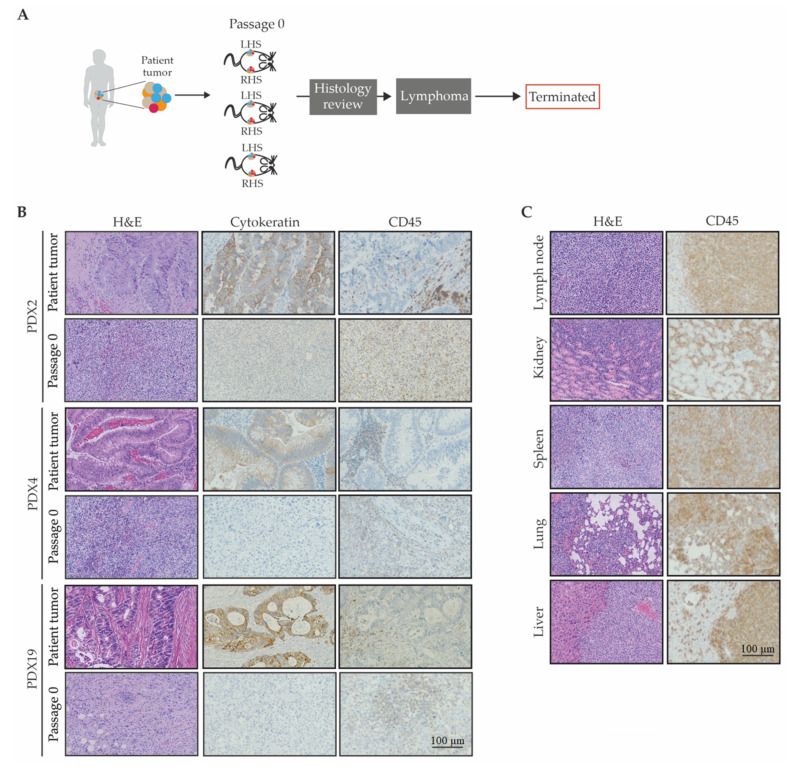
Histological characterization of leukocytic infiltrates in CRC patient-derived xenografts. (**A**) Schematic representation of the histological review of PDXs; (**B**) representative H&E, pan-cytokeratin, and human CD45 immunohistochemistry images of P0 PDX tumors from the indicated PDX line; (**C**) representative H&E and CD45 immunohistochemistry images of metastatic tumors; scale bar for B-C is 100 µm and applies to all images.

Upon histological evaluation of P0 PDX tumors, three PDX lines (PDX2 (neuroendocrine), PDX4, and PDX19) did not resemble the original tumors from which they were derived (Figure 3A). Instead of human epithelial cells, identified by pan-cytokeratin, the PDX tumors contained human leukocytes, indicated by CD45 positive cells (Figure 3B). In particular, these xenograft tumors were populated by mononuclear cells, ranging from small lymphocytes to large atypical lymphoid cells with pleomorphic nuclei and abundant basophilic cytoplasm, consistent with lymphoma.

Upon autopsy of the mice harboring these PDX lines, it was further observed that the human lymphoid cells were also present in the axillary lymph nodes, kidney, spleen, lungs, and liver (Figure 3C). There was no evidence of distant metastases observed in PDX tumors of histologically confirmed epithelial adenocarcinomas at P0, or at any other passage.

Lineage tracking and histological review of left-hand side (LHS) and right-hand side (RHS) xenograft tumors from the twenty-two PDX lines revealed that four PDX lines (PDX1, PDX15, PDX27, and PDX37) had a mixture of lymphoid features in some tumors and epithelial cell content in other tumors (Figure S3A). For these mixed lineage PDX lines, after each passage and histological verification, only the epithelial tumors lines were maintained (Figure S3B). In one of the PDX lines, PDX22, it was observed that a lymphocytic tumor developed on one flank and an adenocarcinoma on the other (Figure S3C). Upon serial transplantation of the tumor that was identified as an adenocarcinoma, it gave rise to a lymphocytic tumor, and as a result, this line was classified as lymphocytic and terminated. In general, the lymphocytic tumors grew faster than the epithelial tumors (Figure S3D). Each of the lymphoid PDX lineages were destroyed, and the epithelial lineages were annotated and separated from the main PDX biobank; however, they remain included in the “successful engraftment” rate of the PDX library. These observations result in a PDX library of 16 adenocarcinomas, 2 adenomas (18/33), resulting in an overall 55% success.

### 2.2. Characterization of Patient-Derived Xenografts

#### 2.2.1. Human Stromal Cells Do Not Engraft with Patient Tumor Tissue

**Figure 4:**
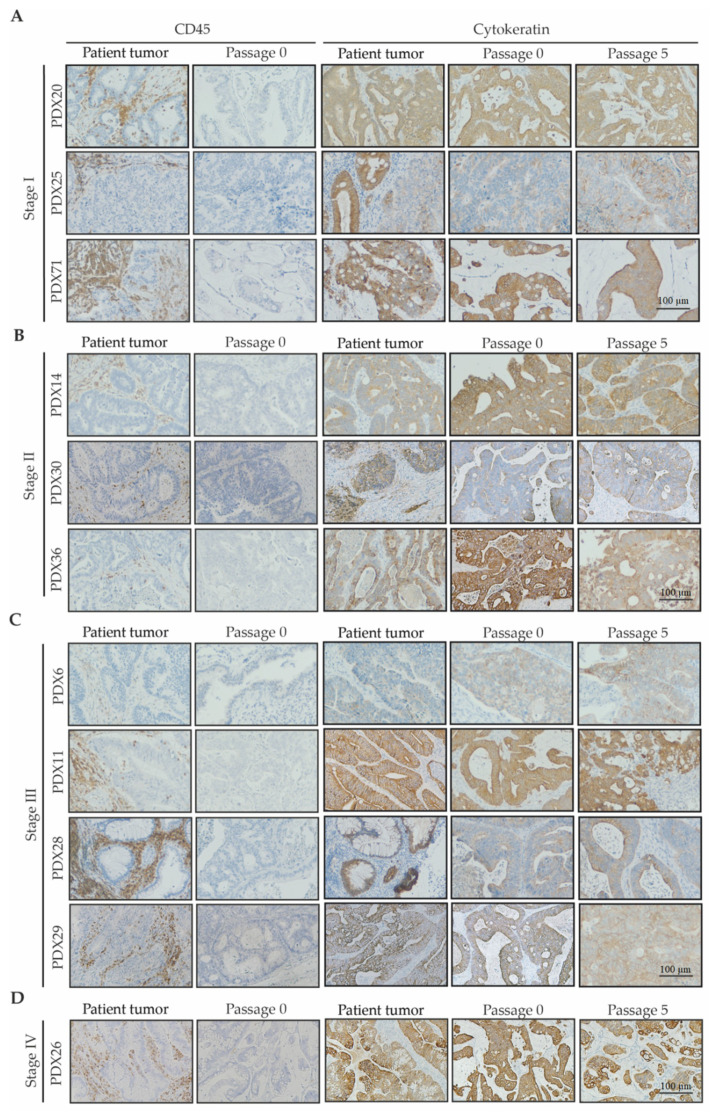
Immunohistochemical characterization of leukocytic infiltrates in CRC patient-derived xenografts. Representative human CD45 and pan-cytokeratin immunohistochemistry images of the patient tumor and indicated passages from the PDX lines from (**A**) stage I; (**B**) stage II; (**C**) stage III; and (**D**) stage IV tumors; scale bar for A-D is 100 µm and applies to all images.

For the PDX lines that successfully engrafted, the components of the tumor stroma were examined to determine if other human cells beyond epithelial cells successful transferred with the patient tumor. For all successful PDX lines, no human CD45 positive leukocytes were present after the first passage (Figure 4A–D). Pan-cytokeratin, a marker of epithelial cells, was expressed by all xenograft tumors confirming successful tumor cell growth, with similar expression levels maintained throughout serial passages (Figure 4A–D). Similar to the loss of immune cells, no human fibroblasts were detected after the first passage of each PDX, with the stromal compartment within the PDX tumors confirmed to be derived from murine fibroblasts (Figure S4A–D), suggesting that human epithelial cells direct murine stromal organization.

#### 2.2.2. Genetic Signatures Are Retained in PDX Tumors during Early Serial Passages

To determine if PDX tumors were genetically representative of the patient tumors from which they were derived, we undertook sequencing using the Accel-Amplicon 56G Oncology Panel. To this end, we compared the genetic profile of two PDX tumors (from P3) to the original patient tumor tissue, and matched patient normal tissue (Figure S5A). Overall, the mutations found in the PDX tumors were consistent with those in the parental tumors, and additional mutations were not observed in the serially transplanted tumors.

As we had anticipated, in all of the tumor tissues, *APC* was altered, consistent with this being one of the first acquired mutations in CRC [53,54]. In PDX15, the *APC* alteration was likely germline, as it was also present in the normal tissue (Figure S5B). Alternatively, the normal tissue may have contained early lesions that were not apparent at the time of surgical resection. Similarly, in all of the tumor tissue, *P53* was altered, consistent with this being another commonly mutated gene in CRC [53,54]. However, *TP53* was also altered in the normal tissue from these patients (Figure S5B).

*FGFR1* was also altered in the patient normal, tumor, and PDX tumor tissue for both PDX15 and PDX36. Fibroblast growth factor receptor (FGFR) 1, which encodes for *FGFR1* is generally reported to be overexpressed in CRC [55] and promotes cell proliferation, cell survival, and angiogenesis, resulting in tumor development [56]. Glutamate metabotropic receptor 1 (*GRM1*) and retinoblastoma 1 (*RB1*) were additional alterations found in PDX15, for the patient normal, tumor, and PDX tumor tissue (Figure S5B). *GRM1* has primarily been implicated in neurodegenerative malignancies but has been reported to promote tumorigenesis [57]; however, few reports exist for *GRM1* involvement in CRC. On the other hand, the loss of *RB1*, a tumor suppressor gene, is common in many cancers [58].

In PDX36, additional alterations found in the PDX tumors were *ABCA12*, *HNF1A*, *KDR*, *KIT,* and *KRAS* (Figure S5B). Frameshift mutations were identified in ATP-binding cassette subfamily member 12 (*ABCA12*), a member of the ABC transporter family and hepatocyte nuclear factor 1-alpha (*HNF1A*), a gene important for epithelial cell growth and cell lineage differentiation. Kinase insert domain receptor (*KDR*, also known as vascular endothelial growth factor receptor 2) is the main mediator of VEGF signaling that promotes tumor angiogenesis [58]. It has been reported to be rarely mutated in CRC [54]. *KIT*, a member of the receptor tyrosine kinase family, is generally associated with increased proliferation, metastasis, and poor prognosis in CRC [59]. Unlike the usual mutations in *KRAS* occurring in codons 12, 13, or 61, an inactivating mutation was identified in codon 117.

#### 2.2.3. Serial Passaging Increased the Successful Growth of Individual Tumors

**Figure 5:**
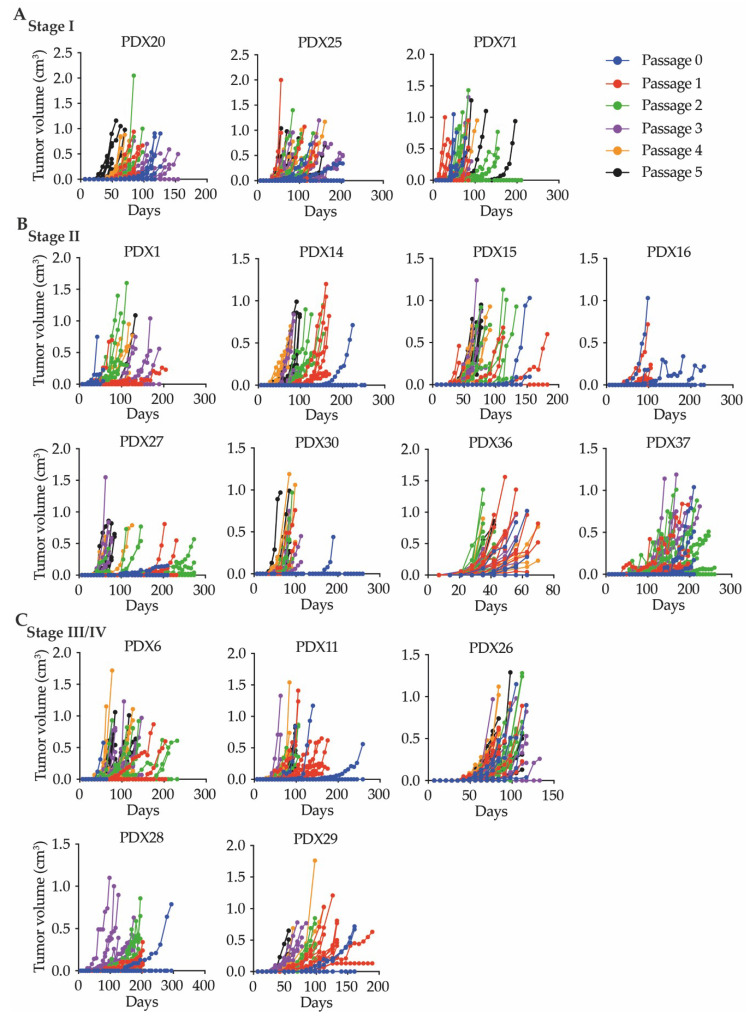
Patient-derived xenograft tumor growth. Passages are indicated in different colored symbols, with P0 (red), P1 (blue), P2 (green), P3 (purple), P4 (orange), and P5 (black). Each line represents an individual tumor. Data are presented for at least two mice per passage, with two tumors per mouse. PDX lines are grouped based on the stage of the parental tumor (**A**) stage I; (**B**) stage II; and (**C**) stage III/IV.

There was considerable variability between the ‘’take rate’’ of an individual piece of tumor tissue at P0, which ranged from 16% to 100%. We have defined the ‘’take rate’’ as the successful growth of an original or serially passaged PDX tumor. However, we observed that variability in the ‘’take rate’’ decreased following serial transplantation. Analysis of the latency of PDX tumors, defined as the time required for a tumor to be palpable, showed that most tumors were palpable in less than 100 days, with decreases in tumor latency with increasing passage (Figure S6A–C). The growth rate, defined as the time required for an individual PDX tumor to reach 1cm^3^, also varied considerably between PDX lines (Figure 5A–C). Additionally, there was variability within the same passage of some PDX lines, and within the different passages of all PDX lines. This variability in growth pattern was not associated with the stage of the parental tumor, or other patient clinical features (Table S2). Since the PDX tumors displayed variable tumor latency and growth kinetics, the proliferation, indicated by Ki67 positive cells, was examined. However, no obvious differences in Ki67 positive cells were observed between the parental tumors and their associated PDX tumors (Figure S7A–D).

### 2.3. Therapeutic Treatment of Patient-Derived Xenografts

#### 2.3.1. Patient-Derived Xenograft Response to Chemotherapy Reflects the Patient Response

**Figure 6:**
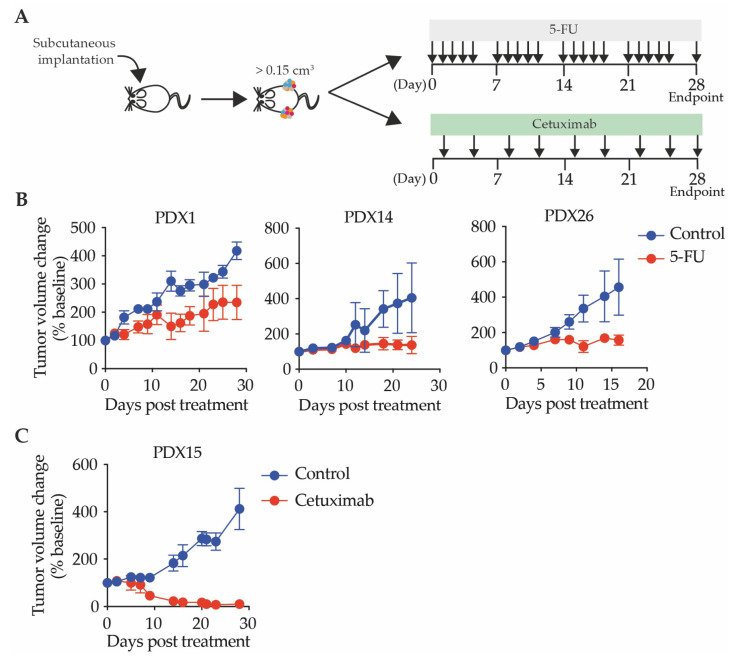
Treatment of patient-derived xenografts. (**A**) Schematic representation of treatment protocols; (**B**) tumor volume following treatment with 10 m/kg 5-FU for PDX1 or 15 mg/kg for PDX14 and PDX26; and (**C**) tumor volume following treatment with cetuximab 20 mg/kg. Data are represented as mean ± SEM with at least two mice per passage, with two tumors per mouse.

5-FU-based adjuvant chemotherapy has been used to treat CRC for several decades [45,46,60]. In order to determine if PDXs could be used to model patient response to chemotherapy, three chemotherapy-naive PDX lines (PDX1, PDX14, and PDX26) underwent 5-FU treatment (Figure 6A). We observed a trend towards increased latency after each passage, consistent with previous reports that breast PDX tumors exhibited shorter latency after undergoing sequential treatment with 5-FU, cisplatin, olaparib, and lurbinectedin, suggestive of adaptation to the chemotherapy agents [61]. In order to assess the impact of 5-FU treatment on tumor growth, the percent tumor volume change relative to the volume at the start of treatment was measured for each passage. In all instances, 5-FU treated PDX tumors grew more slowly than untreated PDX tumors (Figure 6B). The patient from which PDX1 was derived received FOLFOX (5-FU and oxaliplatin) as a first line treatment, and a different second line treatment due to minimal treatment response, which is reflected in the minor response to 5-FU observed for the chemotherapy naive PDX. The patient from which PDX14 was derived did not receive a 5-FU-based treatment, while the patient from PDX26 responded to a combination of 5-FU, oxaliplatin, and leucovorin post-surgery, consistent with the PDX response to 5-FU.

#### 2.3.2. Patient-Derived Xenografts Can Be Utilized to Test Response to Targeted Therapies

Monoclonal antibodies that target the epidermal growth factor receptor (EGFR) are an approved targeted therapy for chemotherapy refractory CRC patients that do not harbor mutations in *KRAS* [62,63]. We treated the chemotherapy-naive PDX15, which was *KRAS* wild-type, with cetuximab as a monotherapy, and observed a reduction in tumor burden (Figure 6C), consistent with the predicted patient outcome based on clinical criteria for cetuximab treatment. The patient from which this PDX was derived did not receive treatment after surgery. Taken together, these results highlight the potential for our PDX biobank to inform pre-clinical therapeutic studies.

## 3. Discussion

Historically, human CRC cell lines have been used in vitro and in vivo for pre-clinical drug trials; however, cell lines only partially recapitulate the molecular diversity, genetic signature, and heterogeneity of CRC tumors [3,64]. PDX models have been reported to more accurately reflect the patient tumors from which they were derived in terms of morphology, tissue architecture, and mutational stability [65]. PDX models are now considered to provide a superior experimental model system of pre-clinical drug responses [66,67,68]. Here, we describe the generation of a PDX biobank from stage I-IV chemotherapy-naive CRC tumors.

The overall success rate of our PDX biobank is comparable to other studies (58.5% [36]; 60% [37]; and 63.5% [38]), although there is considerable variability in reported success rates (87% [39], and 87.5% [40]). Multiple factors may contribute to these discrepancies, including the under reporting of lymphocytic transformation as a PDX failure. In our PDX library, 8 of the 22 PDXs (36%) developed a lymphocytic phenotype, in line with previous reports of the development of Epstein Barr virus (EBV)-associated B-cell tumors in 52% (11/21) of PDXs [69]. While our lymphocytic PDXs were positive for human CD45+, we have not confirmed whether they were B or T-cells. The formation of lymphocytic PDX tumors after implantation has been reported for gastric, breast, ovarian, and lung biobanks [70,71,72], including metastatic lymphocytic lesions [72]. Previous studies also report a strong association between aggressive CRC tumors from patients with metastatic tumors, lymph node metastasis, and poor differentiation and PDX success [38,40]; however, we found no correlation between PDX success and TNM staging of the primary tumor. Our study supports the notion that variations in tissue bulk, technical challenges of tissue collection, and tumor initiating cell content within transplanted samples likely contribute to variability in PDX success rates [73,74].

Like other CRC PDXs, our CRC PDXs were found to harbor wild type *KRAS*, which is not common in traditional CRC cell lines, which will be highly advantageous for pre-clinical studies in cetuximab resistance, for example [38,75,76]. The ability of human tumor cells in PDX models to adapt to their murine host environment is a characteristic shared across numerous tumor types [77]. Conflicting results have been reported with respect to when the human stroma is replaced by mouse stroma. Some studies suggest that murine stroma replaces the human stroma immediately after tumor implantation [38,78,79,80], consistent with our observations. These studies demonstrated that human stromal cells were incapable of proliferating, determined by the absence of proliferating cell nuclear antigen (PCNA) immunostaining [78,79,81]. However, other studies suggest that when human CRC tumor cell suspensions were injected subcutaneously, instead of tumor fragments, that the human stromal cells were still present in P1 [80] and P2 [82]. However, murine stromal cells have been reported as early as P0, the initial transplant, with the increase in murine stroma proportional to the tumor mass [52]. The interaction of human tumor cells with the surrounding stromal cells is important for the retention of the tissue architecture of the parental tumor after implantation [83], and may impact treatment response if murine signals can feedback onto tumor cells.

Our study highlights that CRC PDXs are a robust model system that resembles the parental tumor from which it was derived. We have utilized this biobank to demonstrate that the growth of tumors was reduced after 5-FU treatment, suggesting that perhaps slow-growing tumor clones were selected for, as previously observed in ovarian cancer PDXs [84,85]. Our PDX biobank could also be utilized to model intratumor heterogeneity and clonal selection in future studies, which commonly occur in CRC [86,87,88] and may influence chemotherapy response. Similar to other studies, our CRC PDX tumors match patient response to 5-FU [37,38,89,90,91], and can be utilized to understand response to targeted therapies, like cetuximab, an anti-EGFR antibody [37]. As such, this PDX biobank will unquestionably contribute to the pre-clinical characterization of numerous new therapeutics, and combinations thereof.

## 4. Materials and Methods

### 4.1. Establishment of Patient-Derived Xenografts

#### 4.1.1. Human Tissue Collection

Tumor and adjacent non-tumor tissue were obtained from 33 deidentified treatment naïve patients who underwent surgical tumor resections at the Royal Melbourne Hospital, Box Hill Hospital, the Austin Hospital, or Peter MacCallum Cancer Centre in Melbourne Australia, between February 2015 and May 2017. Written consent from each patient was obtained by the Victorian Cancer Biobank (VCB), in accordance with protocols approved by the Human Research Ethics Committee (HREC) at the Walter and Eliza Hall Institute of Medical Research (WEHI; HREC approval #14/15).

Information on the tumor type and staging was provided by the treating hospitals’ pathology department. Additional deidentified patient details including patient age, gender, tumor site, histologic grade, TNM classification, and differentiation grade were provided by the VCB (project #14019).

#### 4.1.2. Tissue Processing

Fresh tumor and adjacent normal tissue were collected in separate tubes containing transfer media (RPMI; Thermo Fisher Scientific, Waltham, MA, USA containing 10% (*v*/*v*) faetal bovine serum (FBS); Sigma-Aldrich, St. Louis, MO, USA, F9423) and delivered to the laboratory on ice. Tissue was cut into multiple 2 × 2 millimeter (mm) fragments. Two normal and two tumor fragments were snap frozen and archived. One normal and one tumor fragment were fixed in 10% neutral-buffered formalin (Australian Biostain, Victoria, Australia, ACFG) at room temperature (RT) for histopathological analyses. Two normal and two tumor fragments were transplanted into individual mice. The remaining tumor fragments were cryopreserved in 90% (*v*/*v*) FBS (Sigma, F9423) containing 10% (*v*/*v*) dimethyl sulfoxide (DMSO; Sigma, D2650).

#### 4.1.3. Engraftment of Human Colorectal Cancer Tissue

NOD scid IL2R gamma null (NSG) mice were bred and maintained in a specific pathogen free (SPF) animal facility at WEHI, and all procedures were approved by the WEHI Animal Ethics Committee (AEC approval #2014.017, #2017.033). Prior to engraftment, the tumor tissue was washed twice in cold, sterile Dulbecco’s phosphate buffered saline (PBS; Gibco, Waltham, MA, USA 14190250) solution. The recipient NSG mice were anaesthetized through isoflurane inhalation. The dorsal side of the upper flank of the mouse was cleaned with 80% (*v*/*v*) ethanol, and the fur was parted to make a small 5 mm incision in the skin with a pair of sterile surgical scissors. A small 2 × 2 mm tumor fragment was placed subcutaneously (s.c.) under the skin on contralateral flanks, and the open wounds were closed using surgical wound clips (Clay Adams, Victoria, Australia, 427631), which were removed 10 days after the surgery. Prior to the procedure, the mice received a s.c. injection of the analgesic, Carprofen (10 mg/kg bodyweight: Sigma-Aldrich, St. Louis, MO, USA SML1713). For each patient, tumor tissue was transplanted into at least 3 mice.

#### 4.1.4. Monitoring of Patient-Derived Xenograft Tumor Growth

Tumor growth was monitored once a week by recording the digital caliper measurement of the length diameter (*l*) and width diameter (*w*) of the tumor in mm for up to 6 months. The tumor volume was calculated using the following formula:

(1) $$\frac{pi}{2}\frac{\left(l\;X\;w\right)2}{1000}$$

in Microsoft Excel (Microsoft). When the tumor volume reached 1 cm^3^, animals were ethically euthanized, and the tumors excised for serial transplantation. If no tumor growth was visible in that timeframe, the PDX was deemed unsuccessful.

#### 4.1.5. Therapeutic Treatment of Patient-Derived Xenografts

PDX tumors were resected from passage (P) 1 and transplanted subcutaneously into six-week old NSG mice to generate a cohort termed P2. Once the P2 tumors reached 0.15–0.25 cm^3^, mice were randomly allocated to treatment groups receiving either vehicle (PBS) or 5-FU (Sigma-Aldrich, St. Louis, MO, USA F6627) by intraperitoneal (i.p.) injection for five consecutive days followed by two days of recovery. For mice that received cetuximab, treatments were given by i.p. twice weekly, at 20 mg/kg. The therapeutic treatment continued for 28 days, with tumor volume measured and presented as a percent change from the starting volume.

### 4.2. Histological and Immunohistochemical Analysis

#### 4.2.1. Tissue Fixation and Embedding

Following fixation in neutral-buffered formalin for approximately 24 hours at RT, tissue was embedded in paraffin, and 3 µm thick sections transferred onto superfrost slides (Trajan Scientific Australia Pty Ltd, Victoria, Australia, TJ471042202) for Haematoxylin & Eosin (H&E) staining or immunohistochemical analysis.

#### 4.2.2. Histopathological Analysis of Patient-Derived Xenograft Lines

The morphology of the original patient tumor was compared to different passages of their corresponding PDX lines by a certified clinical pathologist (Dr. Michael Christie; The Royal Melbourne Hospital, RMH) based on percent tumor area, level of infiltration, and percent necrotic area.

#### 4.2.3. Immunohistochemistry

Paraffin sections were dewaxed in xylene and rehydrated in a gradient series of ethanol. Heat-induced antigen retrieval was performed by incubating slides in either citrate buffer (10 mM, pH 6; Thermo Fisher Scientific, Waltham, MA, USA AP-9003-500) or Tris-EDTA-Tween 20 buffer (pH 9, 10× buffer) in a pressure cooker for 15 minutes. After cooling to RT, slides were washed in MQ-H_2_O followed by a 20-minute incubation in 3% (*v*/*v*) hydrogen peroxide (Thermo Fisher Scientific, BSPA5.500) to inactivate endogenous peroxidases. Slides were rinsed in MQ-H_2_O followed by a wash in Tris-buffered saline with 0.1% (*v*/*v*) Tween 20 (TBS-T). Sections were circled with a hydrophobic pen (Thermo Fisher Scientific, 008899) and incubated with 5% (*v*/*v*) normal goat serum (Southern Biotech, Birmingham, AL, USA, 0060-01) in a humidified chamber for 1 hour at RT to block for nonspecific antigens. Following blocking, the sections were then incubated with the appropriate primary antibodies (Cytokeratin (Abcam, Cambridge, UK, Ab27988); CD45 (Cell Signaling, Danvers, MA, USA, 13917S); Ki67 (Abcam, Ab15580); Vimentin (Leica Biosystems, Victoria, Australia, NCL-L-VIM-V9); Vimentin (Abcam, Cambridge, UK, Ab92547)) diluted in blocking buffer in a humidified chamber overnight at 4 °C. The following day, slides were washed in TBS-T for 10 minutes followed by incubation, either in biotinylated secondary antibody or HRP-conjugated secondary antibody (Dako. Santa Clara, CA, USA, P0447 or P0448) in a humidified chamber for 1 hour at RT. The slides were then washed in TBS-T. For biotinylated secondary antibodies, slides were further incubated in streptavidin-HRP conjugate (Vector Laboratories, Burlingame, CA, USA, PK6101) for 30 minutes at RT in a humidified chamber. The slides were washed in TBS-T, and 3, 3’-Diaminobenzidine (DAB; Agilent, Santa Clara, CA, USA, K346811-2) substrate was added. Slides were then counterstained with Haematoxylin and coverslipped.

#### 4.2.4. Imaging 

All images were taken using an Olympus BX43 light microscope with an Olympus DP72 camera using CellSens standard software and saved in TIF format.

### 4.3. Statistical Analysis

All graphs and statistical analysis were generated using GraphPad Prism 8 software. Unless otherwise stated, data are presented as mean ± SEM. Pairwise comparisons were performed using unpaired t-tests, and multiple comparisons were performed using ordinary one-way ANOVA with Tukey’s test. *p*-values less than 0.05 were considered to be statistically significant, with *, **, ***, and **** indicating *p* < 0.05, *p* < 0.01, *p* < 0.001, and *p* < 0.0001, respectively.

## 5. Conclusions

We have successfully established and characterized a biobank of CRC PDXs that can be utilized to identify new treatment opportunities.

## Supplementary Materials

The following are available online at https://www.mdpi.com/2072-6694/12/9/2340/s1, Figure S1. Multiple hospital sites contributed tissue for the generation of PDXs, Figure S2. Histological characterization of CRC patient-derived xenografts, Figure S3. Identification of lymphocytic tumors, Figure S4. Immunohistochemical characterization of fibroblast infiltrates in CRC patient-derived xenografts, Figure S5. Genetic characterization of PDX tissue, Figure S6. Patient-derived xenograft tumor latency, Figure S7. Immunohistochemical characterization of proliferation in CRC patient-derived xenografts, Table S1. Characteristics of the patients from with PDXs were derived, Table S2. Summary of the PDX success rates in the context of patient characteristics.
