# Supplementary material for: A Biobank of Colorectal Cancer Patient-Derived Xenografts
Source: Cancers (Basel). 2020 Aug 19;12(9):2340. doi: 10.3390/cancers12092340 (PMC7563543; doi:10.3390/cancers12092340)
Supplement: Supplementary file 1 [file cancers-12-02340-s001.pdf]

Supplementary Materials

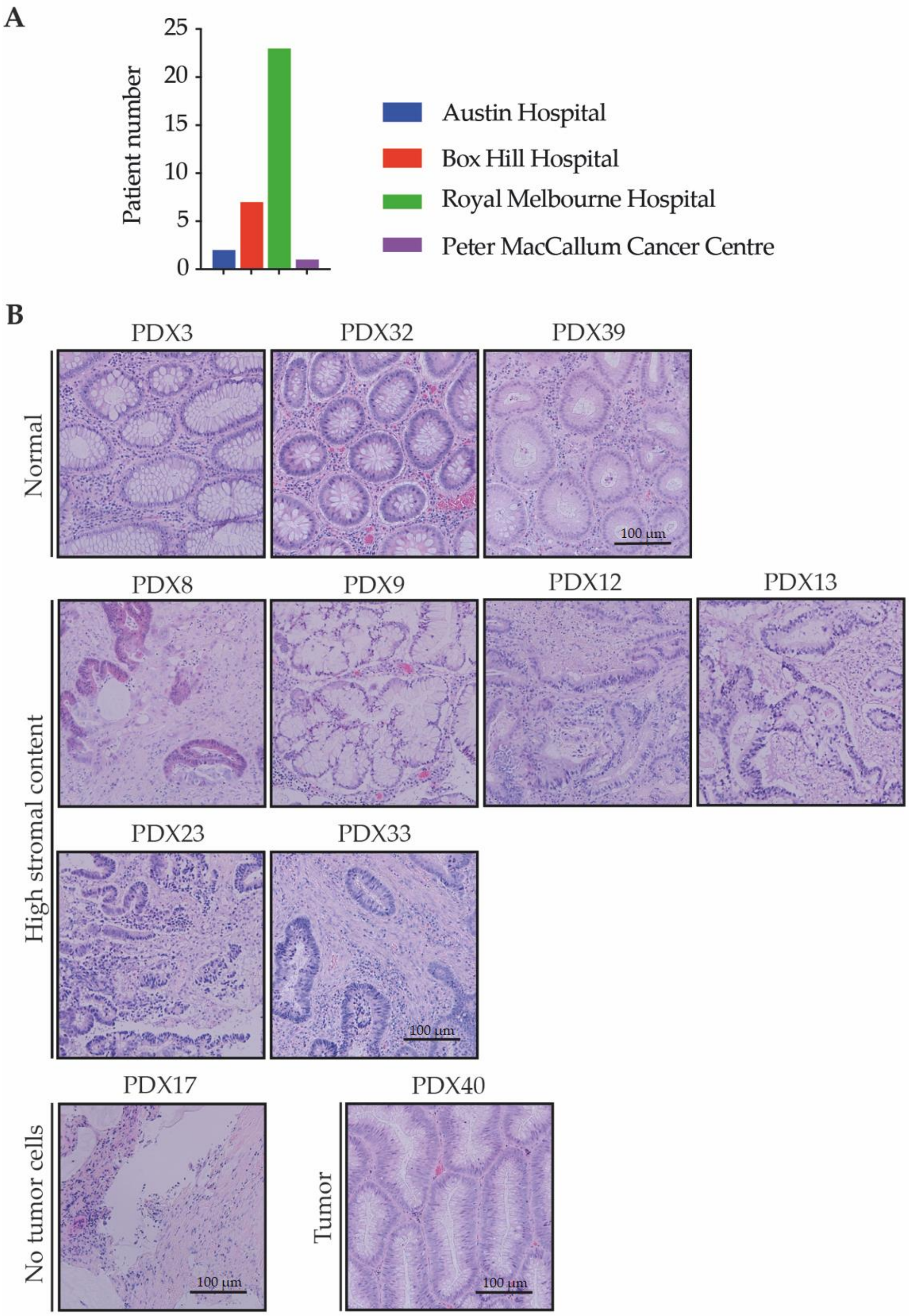

Figure S1. Multiple hospital sites contributed tissue for the generation of PDXs. (**A**) The patient sample distribution according to collection sites. Samples were collected from the Austin Hospital (blue), Box Hill Hospital (red), the Royal Melbourne Hospital (green), and the Peter MacCallum Cancer Centre (purple), in Melbourne, Australia between 2015 and 2017; (**B**) Representative H&E images of the original patient tumors that did not successfully engraft following subcutaneous transplantation into NSG mice. Scale bar is 100 µm and applies to all images.

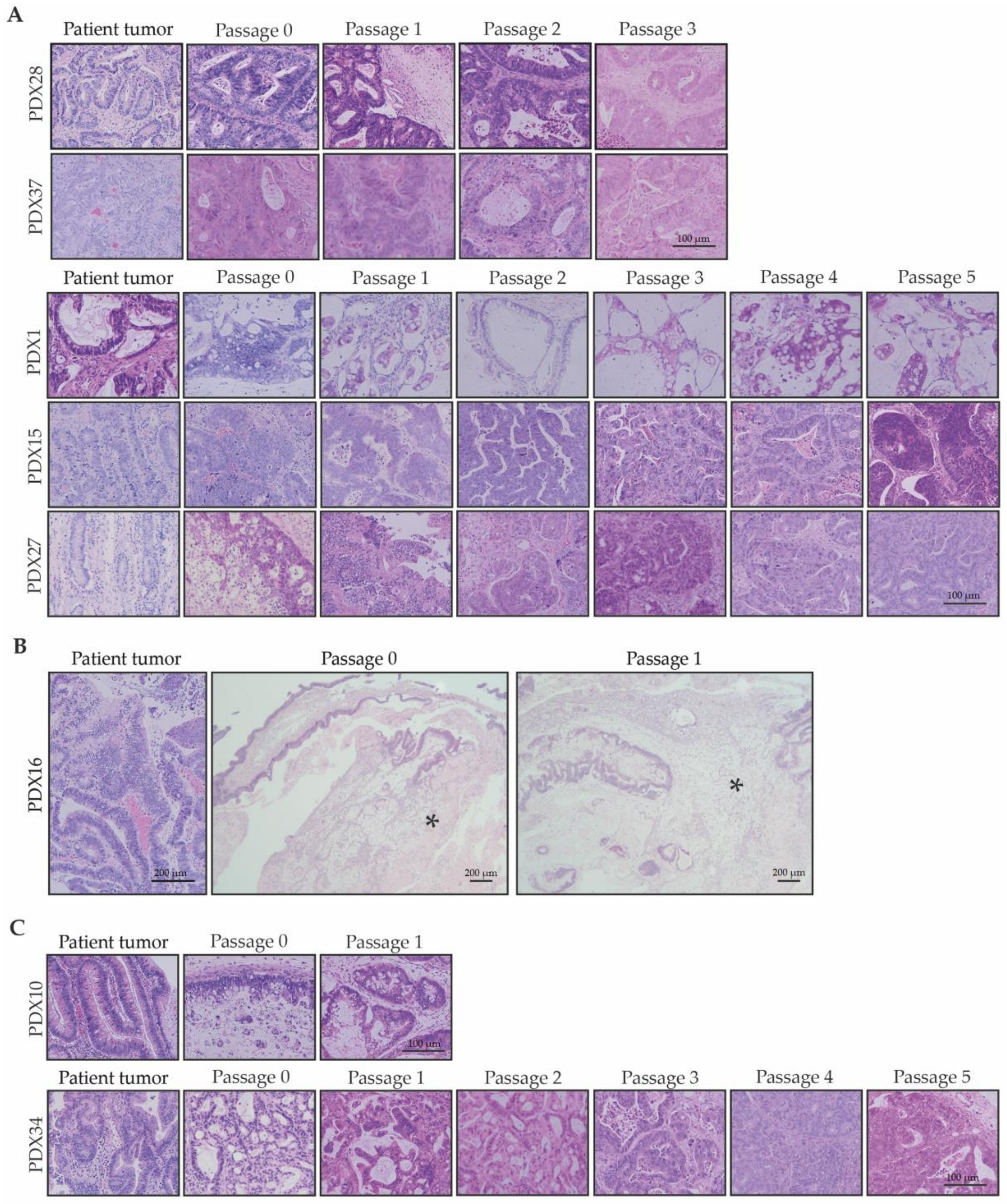

Figure S2. Histological characterization of CRC patient-derived xenografts. Representative H&E images of (**A**) PDX 28 and PDX37, which were passaged four times, whereas PDX1, PDX15 and PDX27 were passaged six times; (**B**) PDX16 was passaged twice. Necrotic core indicated with an asterisk (*); (**C**) PDX10 and PDX34 were adenomas, that were cryopreserved after two and six passages, respectively. Scale bar in **A,C** is 100 µm and applies to all images; apart from (**B**) scale bar is 200 µm and applies to all images.

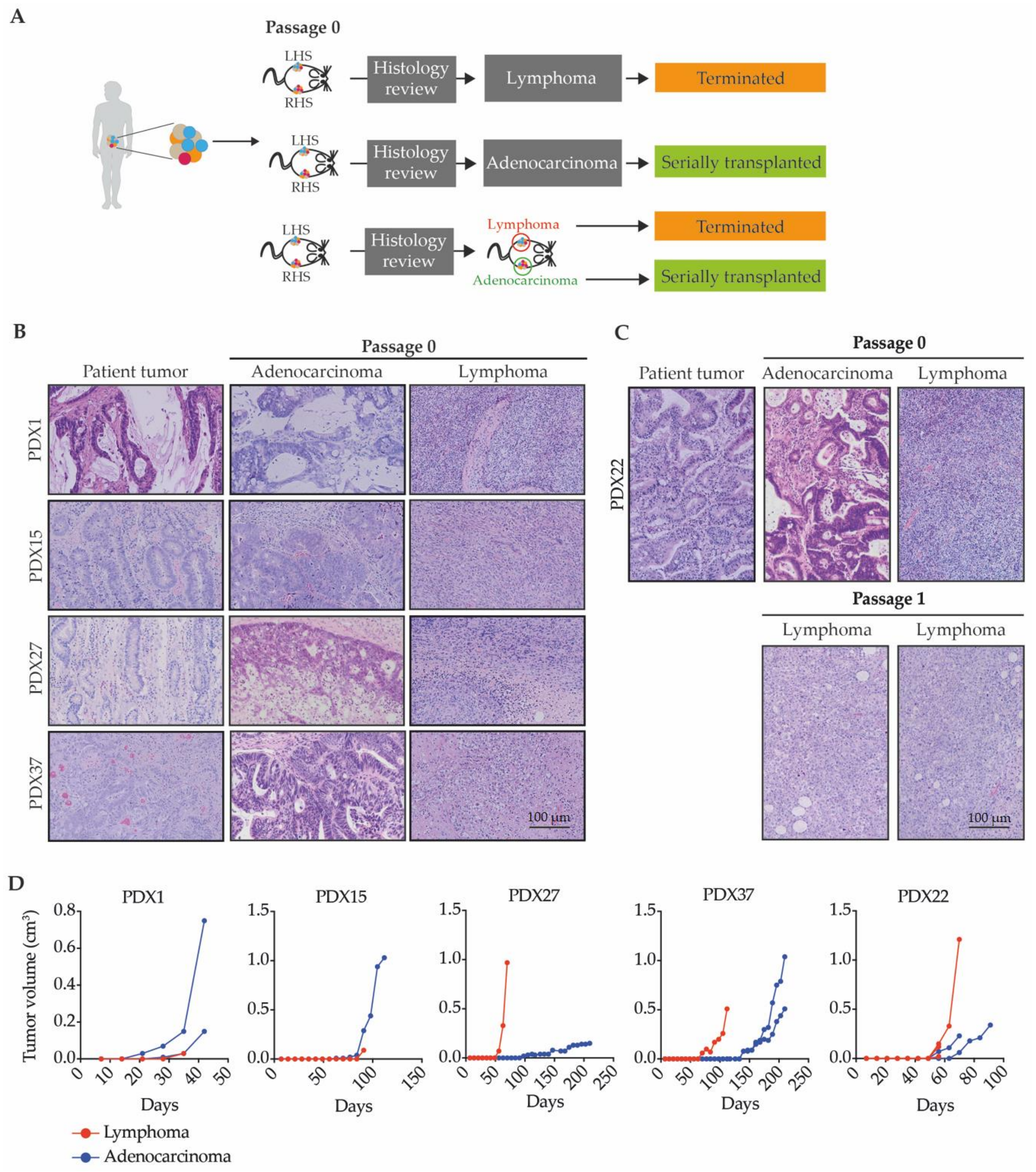

Figure S3. Identification of lymphocytic tumors. (**A**) Schematic representation of lineage tracing of the PDX libraries; (**B**) Representative H&E images of the patient and P0 PDX tumors from the indicated PDX line, where both adenocarcinomas and lymphomas were generated; (**C**) Representative H&E images of the indicated PDX line, where both adenocarcinoma and lymphomas were generated, with the adenocarcinoma subsequently giving rise to lymphomas following passaging; (**D**) Tumor growth of adenocarcinoma (blue) and lymphocytic tumors (red). Scale bar in B-C is 100 µm and applies to all images.

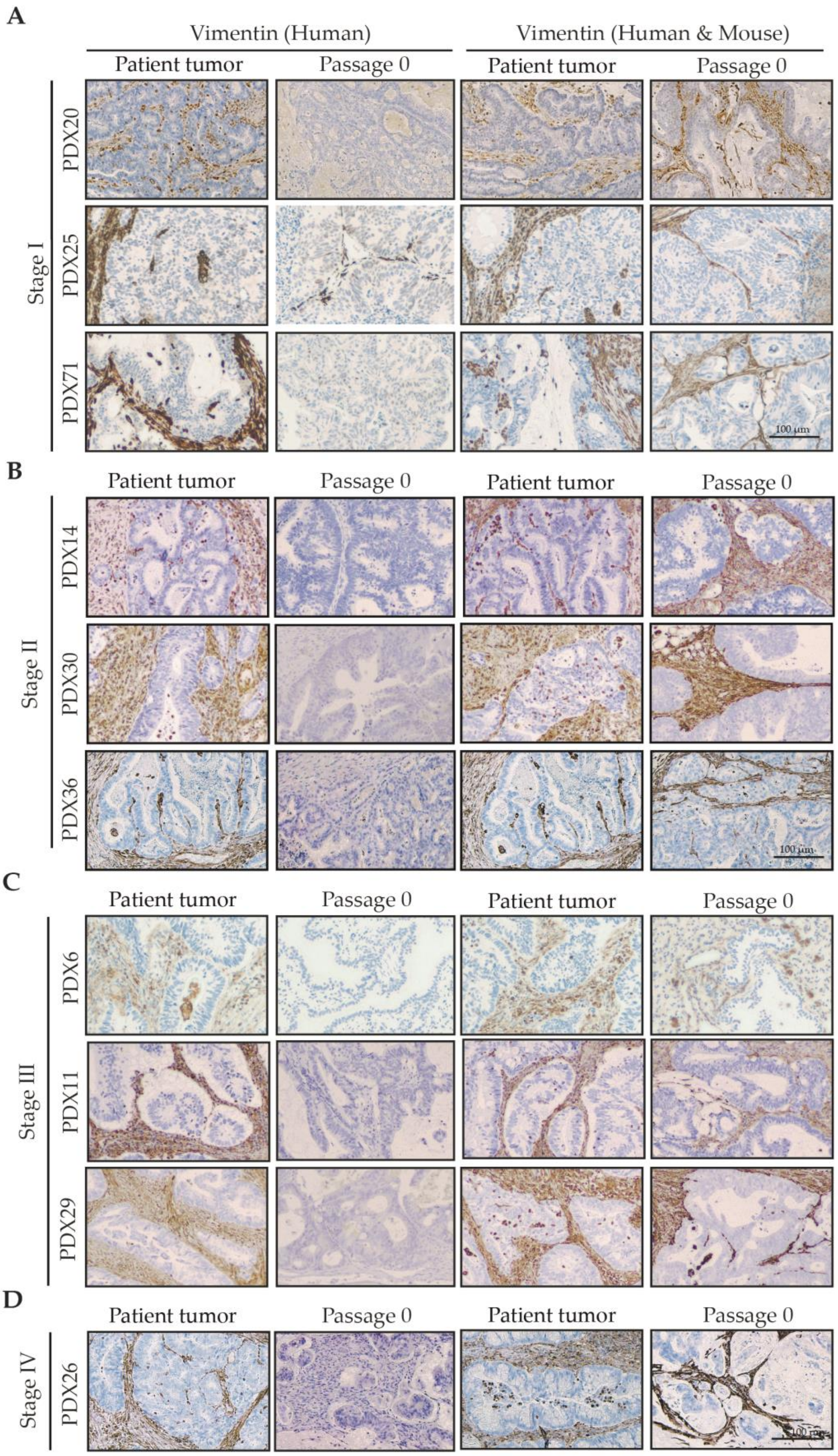

Figure S4. Immunohistochemical characterization of fibroblast infiltrates in CRC patient-derived xenografts. Representative human vimentin and murine vimentin immunohistochemistry images of the patient tumor and indicated passages from the PDX lines from (**A**) Stage I; (**B**) Stage II; (**C**) Stage III; (**D**) Stage IV tumors. Scale bar in **A-D** is 100 μm. and applies to all images.

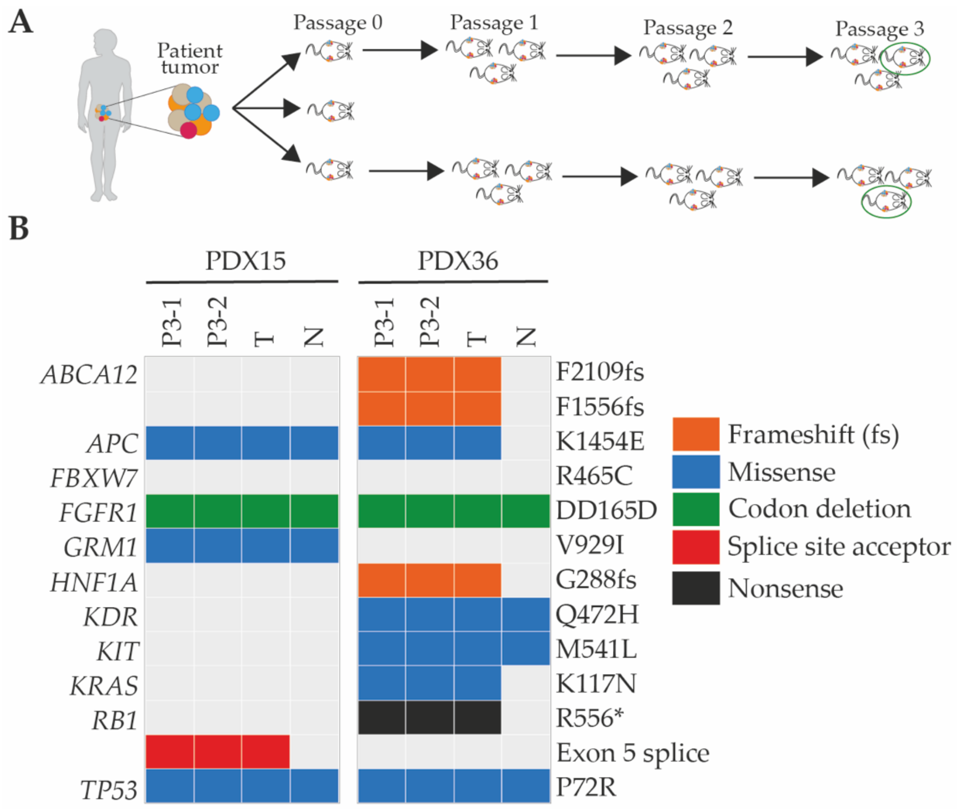

Figure S5. Genetic characterization of PDX tissue. (**A**) Schematic illustration of the samples selected for genomic comparison; (**B**) Alterations identified in key genes for the patient tumor, matched normal tissue, and corresponding PDX tumors from passage 3 (P3) of two different lineages (P3-1 and P3-2) for PDX15 and PDX36, with the genetic alteration indicated by different colors.

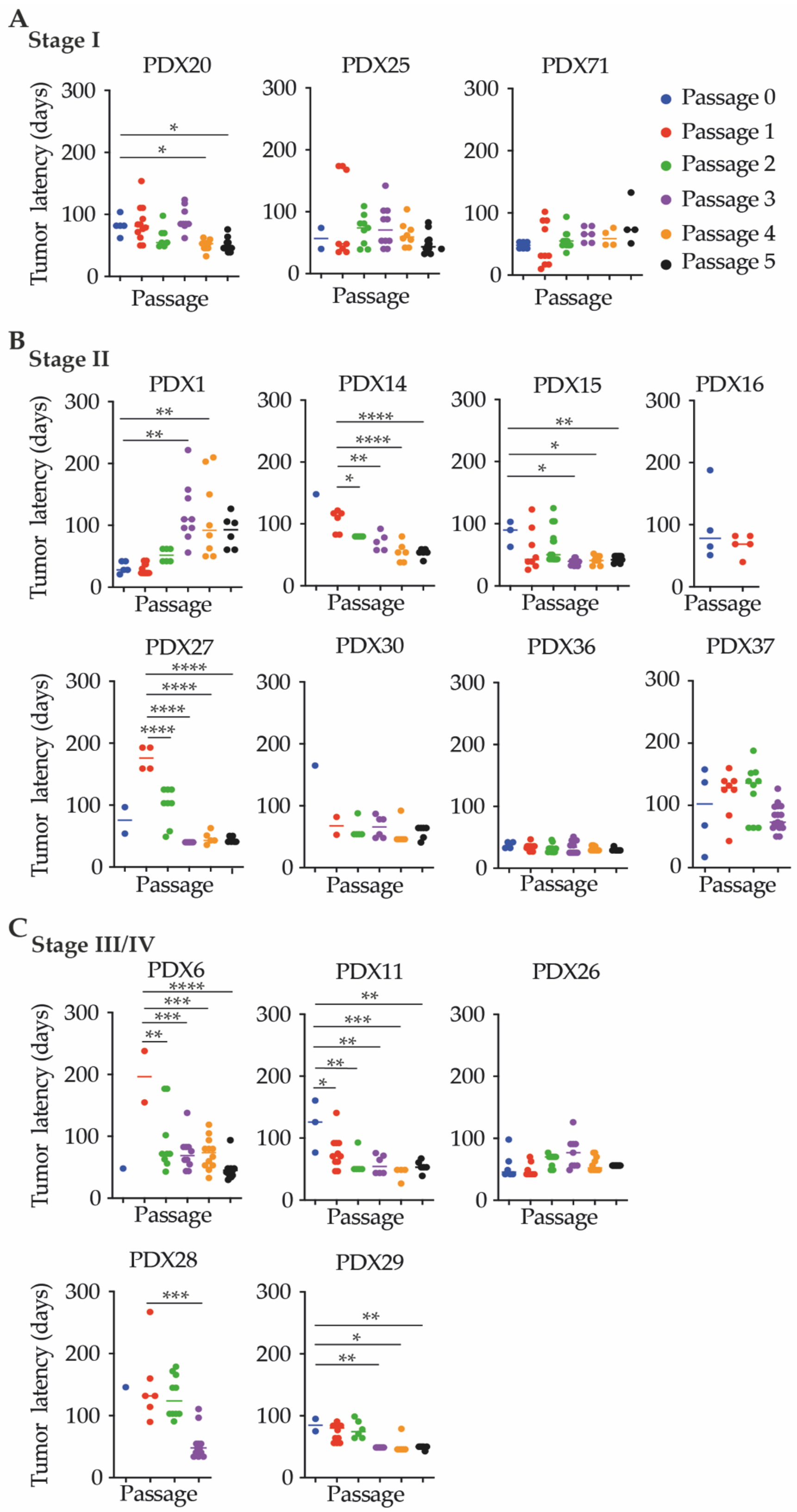

Figure S6. Patient-derived xenograft tumor latency. Passages are indicated in different colored symbols, with P0 (red), P1 (blue), P2 (green), P3 (purple), P4 (orange) and P5 (black). Each symbol represents an individual tumor. Data are represented as mean ± SEM with at least 2 mice per passage, with 2 tumors per mouse. Statistical significance was determined using one-way ANOVA. Statistics are for all passages and are presented relative to P0 or P1. PDX lines are grouped based on the stage of the parental tumor (**A**) Stage I; (**B**) Stage II; and (**C**) Stage III/IV. * $p < 0.05$, ** $p < 0.01$. *** $p < 0.001$, **** $p < 0.0001$.

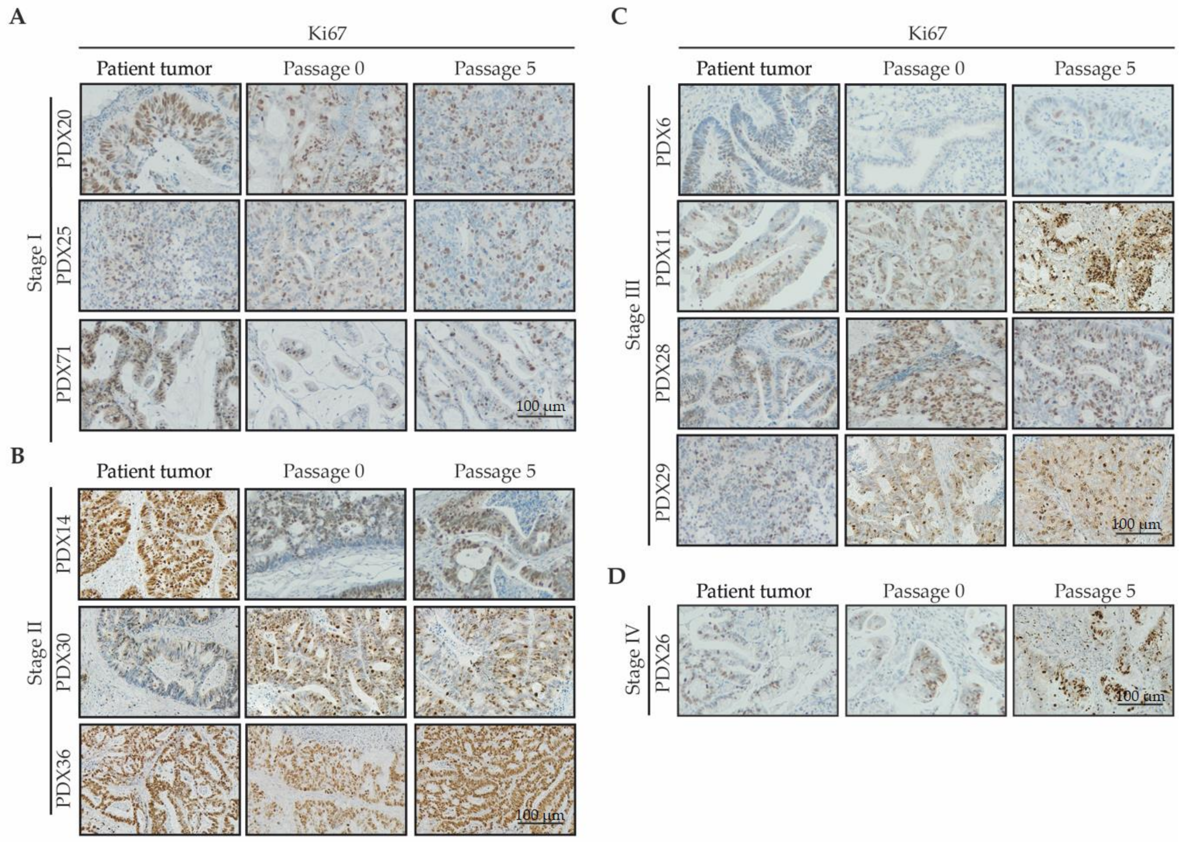

Figure S7. Immunohistochemical characterization of proliferation in CRC patient-derived xenografts. Representative Ki67 immunohistochemistry images of the patient tumor and indicated passages from the PDX lines from (**A**) Stage I; (**B**) Stage II; (**C**) Stage III; (**D**) Stage IV tumors; Scale bar in A-D is 100 µm and applies to all images.

Table S1. Characteristics of the patients from with PDXs were derived.

PDX	Age	Gender	Stage	TNM	Histopathology	PDX Generated
PDX1	65	Female	Stage II	T3N0M0	Moderately differentiated adenocarcinoma with mucinous differentiation	Yes
PDX2	44	Male	N/A	N/A	Neuroendocrine tumor, grade 1	Lymphoma
PDX3	61	Male	Stage I	T2N0M0	Moderately differentiated adenocarcinoma	No growth
PDX4	76	Male	Stage II	T3N0M0	Moderately differentiated adenocarcinoma	Lymphoma
PDX6	26	Female	Stage III	T4N1M0	Moderately differentiated adenocarcinoma	Yes
PDX8	73	Female	Stage II	T3N0M0	Moderately differentiated adenocarcinoma	No growth
PDX9	64	Male	Stage III	T1N1M0	Moderately differentiated adenocarcinoma	No growth
PDX10	79	Male	N/A	N/A	Tubovillous adenoma	Yes
PDX11	77	Female	Stage III	T3N2M0	Moderately differentiated adenocarcinoma	Yes
PDX12	78	Female	Stage II	T3N0M0	Moderately differentiated adenocarcinoma	No growth
PDX13	62	Female	Stage II	T3N0M0	Moderate to poorly differentiated adenocarcinoma	No growth
PDX14	55	Male	Stage II	T3N0M0	Moderately differentiated adenocarcinoma	Yes
PDX15	57	Male	Stage II	T3N0M0	Moderately differentiated adenocarcinoma	Yes
PDX16	66	Male	Stage II	T3N0M0	Moderately differentiated adenocarcinoma	Yes
PDX17	58	Male	Stage II	T3N0M0	Well differentiated mucinous adenocarcinoma	No growth
PDX19	75	Male	Stage III	T3N1M0	Moderately differentiated adenocarcinoma	Lymphoma
PDX20	71	Female	Stage I	T3N0M0	Poorly differentiated adenocarcinoma	Yes
PDX22	66	Male	Stage I	T2N0M0	Moderately differentiated adenocarcinoma	Lymphoma
PDX23	73	Female	Stage II	T3N0M0	Moderately differentiated adenocarcinoma	No growth
PDX25	32	Female	Stage I	T2N0M0	Moderately differentiated adenocarcinoma	Yes
PDX26	75	Male	Stage IV	T4N2M1	Moderately differentiated adenocarcinoma	Yes
PDX27	72	Male	Stage II	T3N0M0	Moderately differentiated adenocarcinoma	Yes
PDX28	34	Female	Stage III	T3N2M0	Moderately differentiated adenocarcinoma	Yes
PDX29	67	Male	Stage III	T3N1M0	Moderately differentiated adenocarcinoma	Yes
PDX30	70	Male	Stage II	T3N0M0	Moderately differentiated adenocarcinoma	Yes
PDX32	62	Male	Stage I	T2N0M0	Moderately differentiated adenocarcinoma	No growth
PDX33	57	Male	Stage III	T3N1M0	Poorly differentiated adenocarcinoma	No growth
PDX34	64	Female	N/A	N/A	Tubovillous adenoma	Yes

PDX36	18	Female	Stage II	T4N0M0	Moderately differentiated adenocarcinoma	Yes
PDX37	77	Male	Stage II	T3N0M0	Moderately differentiated mucinous adenocarcinoma	Yes
PDX39	86	Male	Stage II	T3N0M0	Moderately differentiated adenocarcinoma	No growth
PDX40	73	Female	Stage I	T1N0M0	Moderately differentiated adenocarcinoma	No growth
PDX71	40	Male	Stage I	T3N0M0	Moderately differentiated mucinous adenocarcinoma	Yes

Table displaying the PDX ID, age, gender, diagnosis and histology of patient tumors. Overall tumor stage is described for each patient tumor, with the pathologic classification based on the parental tumor sample, which was adjacent to the samples used for PDX establishment and molecular analysis. The initial staging of the disease according to the TNM staging system, which is the pathologic classification where the T category describes primary tumor status; T1: submucosa, T2: tunica muscularis, T3: subserosa, and T4: serosa or other organs. The N category describes the lymph node status, pN0: no malignant lymph nodes, pN+: presence of at least one positive lymph node. M describes distant metastasis, where M0: no metastasis and M+: presence of metastases to at least 1 distant organ. All cases included in this study were staged according to the 7th edition of the AJCC staging manual (2017). Abbreviations: N/A: not applicable.

Table S2. Summary of the PDX success rates in the context of patient characteristics.

Parameters	All (*n* = 30) *	Unsuccessful (*n* = 11)	Successful (*n* = 19)	*p*-Value
Gender, n (%)				0.7116
Female	12 (40)	5 (41.7)	7 (58.3)	
Male	18 (60)	6 (33.7)	12 (66.7)	
Age, n (%)				0.1288
<50	5 (16.7)	0 (0)	5 (100)	
>50	25 (83.3)	11 (44)	14 (56)	
TNM staging, n (%)				0.6721
Stage I/II	22 (73.3)	9 (40.9)	13 (59.1)	
Stage III/IV	8 (26.7)	2 (25)	6 (75)	
Primary tumour (pT), n (%)				0.1413
pT1-pT2	6 (20)	4 (66.7)	2 (33.3)	
pT3-pT4	24 (80)	7 (29.2)	17 (70.8)	
Lymph node (pN), n (%)				0.1562
pN0	22 (73.3)	9 (40.9)	13 (59.1)	
pN+	8 (26.7)	2 (25)	6 (75)	
Distant metastasis (pM), n (%)				1
pM0	29 (96.7)	14 (48.3)	15 (51.7)	
pM+	1 (3.3)	0 (0)	1 (100)	
Differentiation grade, n (%)				0.5366
Well-moderate	27 (90)	9 (33.3)	18 (66.7)	
Moderate-poor	3 (10)	2 (66.7)	1 (33.3)	
Type of tumour, n (%)				1
Adenocarcinoma	26 (86.7)	10 (38.5)	16 (61.5)	
Mucinous adenocarcinoma	4 (13.3)	1 (25)	3 (75)	
Primary tumour location, n (%)				0.2567
Left colon	16 (53.3)	4 (25)	12 (75)	
Right colon	14 (46.7)	7 (50)	7 (50)	

* The two adenoma, and one neuroendocrine tumor are not included in this table. Features of the patients with adenocarcinomas (n = 30). The TNM staging system is the pathologic classification where the T category describes tumour status; T1: submucosa, T2: tunica muscularis, T3: subserosa and T4: serosa or other organs. The N category describes the lymph node status, pN0: no malignant lymph nodes, pN+: presence of at least one positive lymph node. M describes distant metastasis, where M0: no metastasis and M+: presence of metastases to at least 1 distant organ. All cases included in this study were staged according to the 7th edition of the AJCC staging manual (2017). Difference between groups was quantified using Fisher's Exact test, two-tailed.

Supplementary Methods

Genomic characterization of tumor tissue

1.2. DNA Extraction

Snap frozen tissue samples were placed in pre-cooled 2 mL tubes (Eppendorf, Hamburg, Germany 20170-170) containing an individual 5 mm sterile steel bead (Qiagen, Hilden, Germany 69965). RNeasy lysis buffer (RLT, 600 µL) was added and the tissue was homogenised for 2 minutes at 20 Hertz (Hz) using a Tissue Lyser II (Qiagen). The lysate was centrifuged for 3 minutes at 13,000 rpm and the supernatant was removed by pipetting and transferred to an AllPrep DNA spin column placed in a 2 mL collection tube. The column was quickly centrifuged for 30 seconds at 10,000 rpm to filter the solution through the membrane. The wash buffer AW1 (500 µL) was added to the AllPrep DNA spin column and centrifuged for 15 seconds at 10,000 rpm, and the flow-through was discarded. The wash buffer AW2 (500 µL) was added to the AllPrep DNA spin column and centrifuged for 2 minutes at 13,000 rpm to wash the spin column membrane. The AllPrep spin column was placed in

a clean 1.5 mL collection tube and EB buffer (100 µL) was added directly to the spin column membrane and incubated at room temperature for 2 minutes before eluting the DNA by centrifuging for 1 minute at 10,000 rpm. All DNA samples were stored at −80 °C for future analysis.

2.2. DNA Quantification

DNA purity, concentration and integrity were determined using the Nanodrop ND-1000 spectrophotometer (ThermoFisher Scientific) and Tapestation gDNA ScreenTape (Agilent, Santa Clara, CA, USA) Broad-Range Assay). The library was quantified using the Agilent Tapestation and the Qubit™ RNA assay kit for Qubit 2.0® Fluorometer (Life Technologies, Carlsbad, CA, USA).

2.3. Preparation of the Library

Purified DNA was diluted to 5 ng/µL with nuclease-free water and 10 µL was used for targeted sequencing library preparation using Accel-Amplicon™ 56G Oncology Panel (Swift Biosciences, Ann Arbor, MI, USA). An input of 100 ng of genomic DNA was prepared and indexed for Illumina sequencing using the TruSeq DNA sample Preparation Kit (Illumina, San Diego, CA, USA) as per manufacturer's instructions. The indexed libraries were then prepared and diluted to 1.5pM for single end (1× 86 base) sequencing on a NextSeq500 instrument. The base calling and quality scoring were determined using Real-Time Analysis on board software v2.4.6, while the FASTQ file generation and de-multiplexing utilised bcl2fastq conversion software v2.15.0.4.

2.4. Next-Generation Sequencing

Individual samples for the final library were multiplexed at equimolar concentrations. The final library was sequenced on the NextSeq 500 instrument (Illumina, San Diego, CA, USA, USA) using the 150 high output cycle kit v2 chemistry (Illumina) as per the manufacturer's instructions.

2.5. Detection of Mutations

Regions with phred quality scores below 30, and adaptor sequences and reads containing less than 50 base pairs (bp) were excluded. The paired end reads were merged using fast length adjustment of short reads (FLASH) algorithm. Both the flattened and unflattened reads were combined and the adaptor sequences were trimmed. The reads were aligned to the reference genome, HG38 using the Burrows-Wheeler Aligner (BWA) and variants were called using a modified version of Genome Analysis Toolkit (GATK)'s best practice pipeline removing the duplication step and base quality recalibration. Mutations were called using HaplotypeCaller (3.6-0) considering all samples concurrently and annotated using the software, SnpEff.
